# Boron neutron capture therapy combined with immunotherapy: insights from radiotherapy, BNCT-specific evidence, and future directions

**DOI:** 10.3389/fimmu.2026.1687072

**Published:** 2026-05-28

**Authors:** Zeyuan Li, Haolin Cai, Xiukui Cheng, Linping Qi, Xingda Wang, Ting Yang, Can Li, Yumin Li, Cong Chen

**Affiliations:** 1 The Second Hospital & Clinical Medical School, Lanzhou University, Lanzhou, Gansu, China; 2 Gansu Provincial Key Laboratory of Environmental Oncology, Lanzhou, Gansu, China; 3 Department of Orthopedics, The Second Hospital & Clinical Medical School, Lanzhou University, Lanzhou, Gansu, China

**Keywords:** boron neutron capture therapy (BNCT), chimeric antigen receptor T cell therapy (CAR-T), immune checkpoint inhibitors (ICIs), radioresistance, tumor microenvironment remodeling

## Abstract

Boron neutron capture therapy (BNCT), a precision radiotherapy modality, has shown significant promise in targeted cancer treatment. Like conventional radiotherapy, the development of radioresistance hinders the efficacy of BNCT. Nevertheless, the physical parameters in BNCT induce the massive anti-tumor response compared to low-LET radiotherapy. Recent advancements have highlighted its synergistic potential with immunotherapy. In this review, we explore how the unique physical-to-biological trajectory of BNCT drives a potent “Cold-to-Hot” transformation of the tumor microenvironment. We describe how radiotherapy remodels the tumor microenvironment and extrapolate the potential mechanism by which BNCT influences anti-tumor response based on the principles of conventional radiotherapy. BNCT-induced immunogenic cell death and neoantigen presentation activate dendritic cells and CD8^⁺^ T-cells. Besides its immunostimulatory effects, BNCT may also exhibit immunosuppressive effects. Both the recruitment of immunosuppressive cells and tumor hypoxia can influence BNCT efficacy, and studies have shown that targeting these factors can enhance BNCT outcomes. Looking forward, we propose that combining BNCT with CAR-T cell therapy or immune checkpoint inhibitors represents one of the most promising directions for its future application. We hope this review provides novel insights into the clinical translation of BNCT.

## 1 Introduction

Radiotherapy (RT) is currently an important treatment for localized solid tumors, with over half of all cancer patients deriving benefit from it (1). Boron neutron capture therapy (BNCT) is a novel RT approach that relies on nuclear reaction. Specifically, the stable isotope ^10^B captures a thermal neutron, forming an excited compound nucleus ^11^B, which promptly decays by emitting a high linear energy transfer (LET) α particle ^4^He^2+^, and a recoiling ^7^Li nucleus and a small number of γ-rays (2). This process generates high-LET radiation with a cytotoxic range of 5-9 µm, approximately the diameter of a single cell, enabling the preferential damage to cancer cells enriched in ^10^B while sparing adjacent normal tissues lack ^10^B (2). The cell-killing mechanisms of BNCT involve three key components: 1. The high-LET radiation primarily induces direct cellular DNA damage, with potential minor contributions from ROS-mediated oxidative injury, leading to irreversible cell death (3). 2. Radiation-induced immunogenic cell death (ICD) has been reported to contribute to tumor microenvironment (TME) remodeling and may trigger an anti-tumor immune response (4). While nearly all high-LET RT modalities share these fundamental anti-tumor properties, BNCT is distinguished by its tumor-targeting capability and limited cytotoxic range (5) which may mitigate the off-target toxicities associated with conventional RT (6).

Currently, p-boronophenylalanine (BPA) and sodium borocaptate (BSH) are the most widely used boron delivery agents in clinical practice. BPA, an amino acid analogue, enters tumor cells primarily via the L-type amino acid transporter 1 (LAT1), while BSH is a boron cluster that relies on passive diffusion and exhibits high boron content (2). In general, an effective BNCT agent should exhibit high tumor uptake, low intrinsic toxicity, and minimal accumulation in normal tissues. Ideally, the tumor-to-normal tissue (T/N) and tumor-to-blood (T/B) boron concentration ratios should exceed 3:1. In addition, such agents are expected to retain within tumors for several hours while being rapidly cleared from blood and normal tissues (7). However, both BPA and BSH exhibit notable limitations, including suboptimal tumor selectivity, insufficient T/B and T/N ratios, as well as unfavorable pharmacokinetic properties, such as the limited aqueous solubility of BPA and the rapid systemic clearance of both agents. Collectively, these drawbacks may restrict the overall therapeutic efficacy of BNCT (8). To overcome the limitations of conventional agents like BPA and BSH, substantial efforts have recently focused on nanoparticle and nanovesicle-based boron delivery systems (9). These nanoplatforms, including boron-containing nanoparticles, liposomes, and extracellular vesicles, have demonstrated enhanced tumor targeting and improved boron retention (8, 9). Specifically, TME-responsed multifunctional boron drugs and cell-based carriers such as macrophages have emerged as highly promising candidates (4, 10). Notably, some advanced systems enable the co-delivery of immunomodulatory agents, facilitating synergistic interactions between BNCT and immunotherapy (4, 11). However, these promising boron deliveries still remain in the preclinical research phase.

Over the past decades, clinical trials in Finland and Japan have established the safety profile of BNCT in recurrent head and neck cancers and glioblastoma (12, 13), its safety in other malignancies is being validated (14, 15). Although promising clinical outcomes have been reported in multiple BNCT studies, a subset of patients still experiences tumor progression and treatment failure. To address this challenge, combination therapies have garnered considerable attention (16). Notably, RT represents a potential strategy for combination with immunotherapy, primarily due to its ability to remodel the TME (17). Among immunotherapeutic approaches, we consider the combination of BNCT with immune checkpoint inhibitors (ICIs) or chimeric antigen receptor (CAR) T cell therapy to be particularly promising. Especially, several preclinical studies have already reported on the combination of BNCT and ICIs (18). Concurrently, the development of well-targeted boron-based drugs is considered crucial for enhancing the clinical efficacy of BNCT and facilitating its translation into clinical practice (19). Recently, boron drugs conjugated with siPD-L1 have been reported and achieved encouraging results in preclinical experiments (4). Although the combination between BNCT and CAR T cell therapy remains unreported, we will discuss the prospects of such a combination therapy.

In this review, we systematically summarize the current understanding of BNCT-induced TME remodeling and critically evaluate its potential interactions with immunotherapy. In particular, we focus on the emerging rationale for combining BNCT with ICIs and CAR T-cell therapy, while highlighting key knowledge gaps and future research directions for the clinical translation of BNCT-based combination strategies.

## 2 Radiation remodels TME

**Figure 1:**
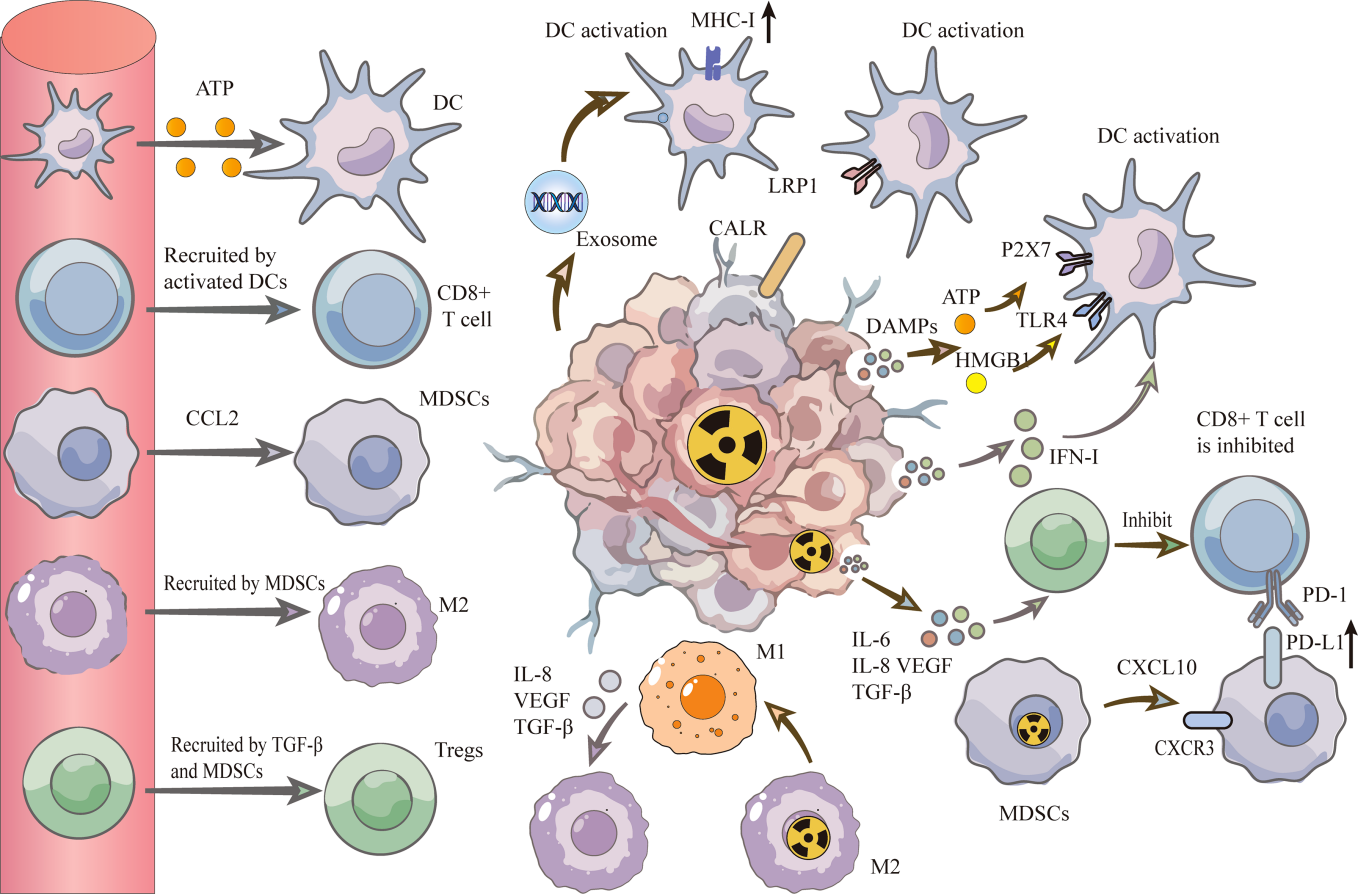
Immune cell migration and cytokine changes in the tumor microenvironment post-radiation. Radiation activates ICD and cGAS-STING signaling pathway, both of which stimulate DCs to promote CD8^+^ T cells infiltration. Upon binding to their respective receptors, ATP, HMGB1, CALR and IFN-I induce immunostimulatory effects. After radiation, tumor cells upregulate neoantigen expression, along with increased MHC-I expression. In response to radiation, M2-type macrophages polarize toward an M1 phenotype, further activating immune activity. Concurrently, tumor cells develop radioresistance in response to radiation by secreting immunosuppressive cytokines, including CCL2, IL-6, IL-10 and TGF-β, to recruit immunosuppressive cells. Among these, MDSCs directly inhibit CD8^+^ T cells via the PD-1/PD-L1 axis, and secrete CXCL10 to recruit more MDSCs, amplifying immune suppression. Additionally, immunosuppressive cytokines drive M1-to-M2 macrophage polarization, exacerbating immune inhibition. Notably, radiation recruits both immunostimulatory and immunosuppressive cells into the TME.

RT can induce tumor immunogenicity by triggering the release of pro-inflammatory or anti-inflammatory mediators and modulating the distribution of infiltrating immune cells in the TME (20). It is well established that RT exerts both immunostimulatory and immunosuppressive effects through multiple mechanisms, which depend on tumor histology, radiation dose fractionation, and host immune status (21). For immunostimulatory effects, numerous pro-inflammatory mediators can convert “cold” tumors (with low immune cell infiltration) to “hot” ones (with high immune cell infiltration) by promoting neoantigen presentation, activating immune effector cells, and inhibiting immunosuppressive cells (17, 22). RT can also induce the regression of non-irradiated metastatic lesions, which is called “abscopal effect” (23). In terms of immunosuppressive effects, chronic interferon signaling is a key contributing factor (24). To avoid excessive inflammatory response, cancer cells secondarily recruit immunosuppressive cells into the TME and develop treatment resistance (25). The main immunosuppressive effects of RT include: 1. Promoting the recruitment of immunosuppressive cells such as Myeloid-derived suppressor cells (MDSCs), Tregs and tumor-associated macrophages (TAMs) (20), 2. Inducing the release of immunosuppressive cytokines including IL-6, IL-10 and TGF-β (17), 3. Exacerbating the hypoxic environment (26), 4. Upregulating the expression of immune checkpoints (27). Targeting these pathways can improve the response to RT, making the combination of RT and immunotherapy a promising strategy (28). **Figure 1** illustrates the potential mechanisms by which RT affects the TME.

As a novel form of RT, BNCT is hypothesized to share some of these immunomodulatory properties (29, 30). This potential was first explored in 2000, a study found that rats treated with both BNCT and cellular immunization exhibited prolonged survival following re-injection with tumor cells one year later (31), suggesting that BNCT may contribute to the induction of long-term immune memory. Additionally, boron drugs loaded with immune enhancers (including immune adjuvants and immunostimulants) have been reported to enhance the immunostimulatory effects associated with BNCT (11, 32, 33). Recent preclinical studies in colon cancer models further support these observations. Specifically, BNCT combined with adjuvants such as oligo-fucoidan and glutamine has been shown to reduce radiotoxicity while enhancing anti-tumor immunity and abscopal effects (32). Moreover, the combination of BNCT with the Bacillus Calmette-Guerin, an immune stimulator, demonstrates synergistic local and systemic anti-tumor efficacy (33).

**Figure 2:**
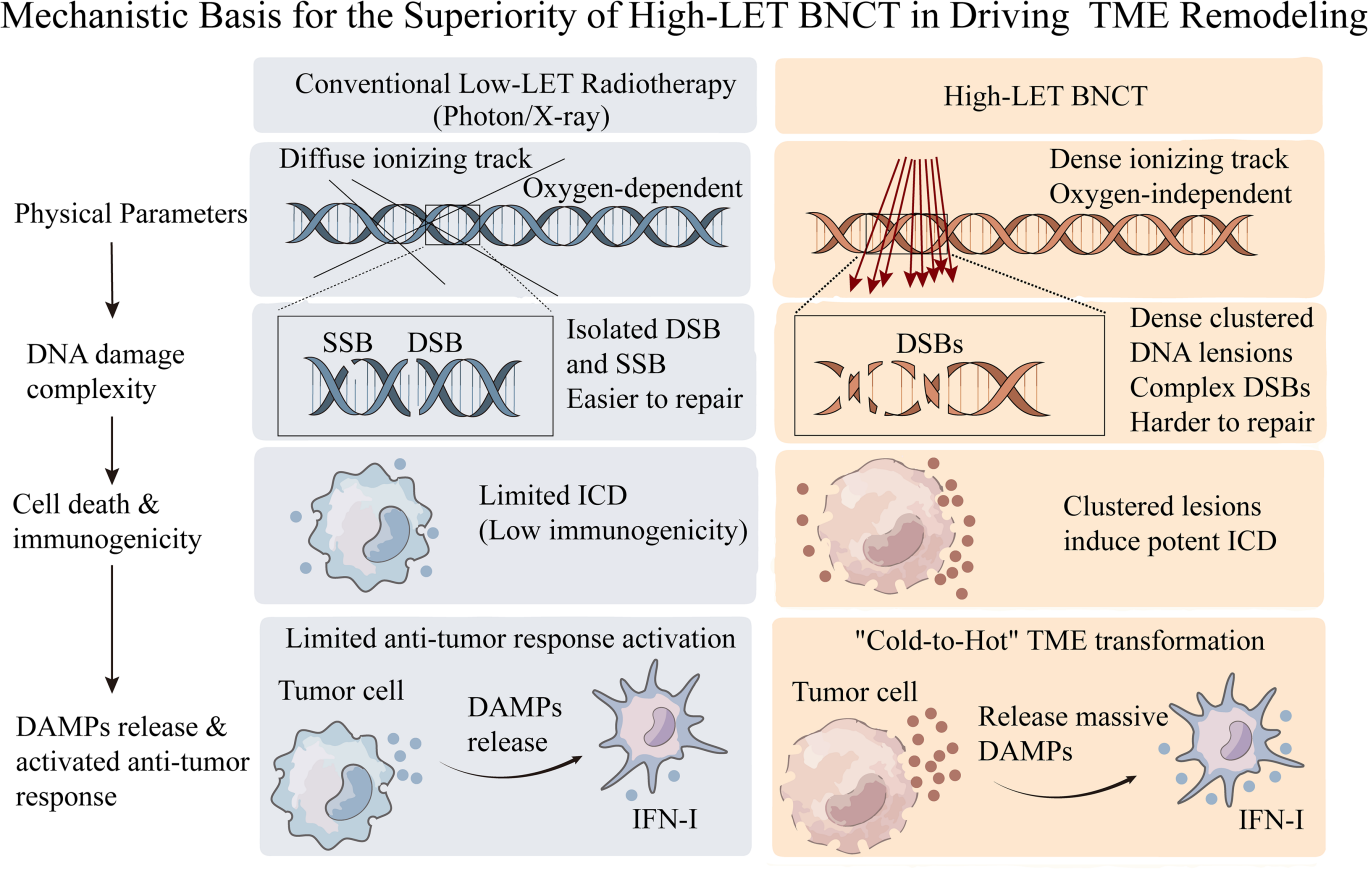
Mechanistic basis for the superiority of high-LET BNCT in driving TME remodeling. Different physical properties between low-LET RT and high-LET radiation induce difference in DNA damage complexity, cell death immunogenicity, and anti-tumor immune activation. Low-LET radiotherapy (photon/X-ray) induces diffuse, oxygen-dependent ionization and forms repairable isolated DNA lesions, leading to limited ICD and restricted anti-tumor responses. In contrast, high-LET BNCT generates dense, oxygen-independent ionizing tracks and induces irreparable clustered DNA lesions, triggering potent ICD, robust DAMP release, and IFN-I production, thereby driving the transformation of immunosuppressive “cold” TME into immune-activated “hot” TME, ultimately potentiating systemic anti-tumor immunity.

The distinctive immunological advantages of BNCT are fundamentally rooted in its superior physical characteristics, which differentiate the biological consequences of high-LET particles from those of conventional photon-based RT (**Figure 2**). High-LET radiations (e.g. BNCT) exhibit exceptional ionization densities that concentrate energy deposition within a localized, micron-scale trajectory (34). In contrast to the sparse and isolated lesions typical of low-LET radiation, high-LET particles traverse the DNA scaffold to induce multiple double-strand breaks (DSBs), single-strand breaks and oxidative base modifications in close spatial proximity, culminating in the formation of “clustered lesions” (35). While the absolute numerical yield of DSBs may only marginally increase with rising LET, the defining therapeutic advantage lies in the surge of damage complexity (36). Such complex clustered lesions are inherently recalcitrant to standard cellular repair machinery—specifically canonical non-homologous end joining (c-NHEJ) and homologous recombination—frequently precipitating significant repair kinetics delays or catastrophic misrepair (34). Ultimately, these physically dictated, irreversible DNA modifications are speculated to contribute to the enhanced ability of BNCT to overcome radioresistance and potentially stimulate anti-tumor response (34). However, direct experimental evidence linking these unique physical characteristics to distinct immunological outcomes (relative to conventional RT) remains scarce and requires further rigorous validation.

## 3 Potential signaling pathways mediated by BNCT

The molecular mechanisms underlying BNCT-induced anti-tumor immune responses remain incompletely understood. A limited number of studies have suggested that BNCT may activate pathways associated with ICD, including the release of damage-associated molecular patterns (DAMPs) and the induction of type I interferon (IFN-I) (37). ICD-associated DAMPs mainly include high-mobility group box 1 (HMGB1), calreticulin (CALR) and ATP (37). Actually, there are limited studies indicating that cGAS-STING and HMGB1 may participate in BNCT-mediated anti-tumor response. However, most mechanistic insights into these pathways are derived from conventional RT and should be interpreted as contextual or hypothesis-generating references rather than direct evidence for BNCT. **Table 1** shows the levels of IFN-1 and HMGB1 in TME following BNCT versus conventional RT.

**Table 1:** Comparison of immunological responses between BNCT and conventional low-LET RT.

Immunological molecular and cellular response indicators	Conventional RT type	Radiation dose	Immunological response profiles of BNCT vs Conventional RT	Reference
HMGB1 level	γ-ray	BNCT: 24Gy-Eq; γ-ray: Equivalent irradiation dose	BNCT: HMGB1 levels increased 14-fold at 24 h compared to 6 h; γ-ray: Levels were more than 2-fold lower than those in the BNCT group	(52)
GM-CSF level	γ-ray	BNCT: 24Gy-Eq; γ-ray: 24Gy	No significant difference	(120)
IFN level	X-ray	BNCT: 4.395 Gy; X-ray: 4 Gy	IFN-α and IFN-β levels in BNCT were significantly elevated than X-ray	(44)
CD8^+^ T cell infiltration	X-ray	BNCT: 4.395 Gy; X-ray: 4 Gy	BNCT: Widespread infiltration within tumors; X-ray: Concentrated in localized tumor regions	
DC function	X-ray	BNCT: 4.395 Gy; X-ray: 4 Gy	BNCT: Stronger antigen presentation capacity; X-ray: Weaker antigen presentation capacity; only mild upregulation of relevant molecules	
TCR expression	X-ray	BNCT: 4.395 Gy; X-ray: 4 Gy	BNCT: Increased expansion proportion of TCR-positive cells; X-ray: Lower expansion proportion of TCR-positive cells	

### 3.1 cGAS-STING

The cyclic GMP-AMP synthase-stimulator of interferon genes (cGAS-STING) signaling axis detects abnormal DNA in the cytoplasm to trigger immune response by producing IFN-I (38). In the context of conventional RT, activation of this pathway has been characterized and contributes to radiation-induced anti-tumor immunity, including the abscopal effect (39). IFN-I is produced by a diverse range of cells, including both immune cells and tumor cells, and potently activates anti-tumor immune reactions (40). It attenuates the function of immunosuppressive cells (specifically reducing the infiltration and proliferation of Tregs and MDSCs in tumors) and enhances the function of immune effector cells (promoting DC differentiation and maturation and increasing the cytotoxicity of NK cells and CD8^+^ T cells) (41). Given these functions, the cGAS-STING pathway has emerged as a potential immunotherapeutic target in anti-tumor immunity. Intriguingly, there is a positive correlation between the intrinsic STING expression in cancer cells and the infiltration levels of CD8^+^ T cells (42). Moreover, clinical study has shown that higher STING expression is associated with a better prognosis for cancer patients who received conventional RT (43).

In contrast, direct and functional evidence for cGAS-STING activation in BNCT remains highly limited, with most findings being phenomenological rather than mechanistic. As shown in **Table 1**, preliminary studies reported elevated IFN-I levels in the TME following BNCT compared to conventional RT in matched tumor models (44). Karimi Roshan et al. found that BNCT application in human microglial cells was associated with increased phosphorylation of STING, and BNCT effectively promoted IFN production and suppressed tumor growth in preclinical models (3, 45, 46). Based on this promising mechanism, Zhang et al. designed BSA-BPA-MnO_2_, a TME-responsive, manganese-enriched nanoboron agent, which triggered a significant anti-tumor response potentially involving activation of the cGAS-STING pathway (47). Although existing evidence suggests that BNCT may engage the cGAS-STING pathway in anti-tumor responses, whether the BNCT-induced cGAS-STING pathway has distinct characteristics compared with other types of RT remains unclear. It is crucial to note that, while BNCT is theoretically more potent in inducing complex DNA double-strand breaks, direct evidence linking specific boron-derived α-particles to cGAS-STING activation is still emerging compared to photon therapy. Formal functional validation, such as STING knockout or genetic silencing in tumor models, is urgently required to clarify the specific regulatory role of this pathway in BNCT.

### 3.2 HMGB1

High mobility group box 1 (HMGB1), a non-histone chromatin-binding protein, translocates from the nucleus to the cytoplasm and is subsequently released into the extracellular space during ICD (48). Extracellular HMGB1 exerts distinct functions upon binding to different receptors. In general, HMGB1 induces chemotactic effects when forming a complex with CXCL12 and binding to CXCR4 (49). In the context of X-ray radiation, HMGB1 exerts immunostimulatory effects when interacting with toll-like receptor 4 (TLR4) (37). TLR4 is highly expressed on innate immune cells, such as DCs. In response to HMGB1, DCs become activated and prime antigen-specific T cells (50). Myeloid differentiation primary response 88 mediates TLR4 signal transduction to induce these immunostimulatory effects (51). Both preclinical study and clinical observations in cancer patients have demonstrated that impaired HMGB1 or TLR4 signaling is associated with reduced anti-tumor immunity and poorer prognosis (51).

In contrast, the role of HMGB1 in BNCT remains incompletely understood. In the context of BNCT, limited preclinical studies have reported elevated HMGB1 levels following treatment, suggesting that HMGB1 may be involved in BNCT-induced immune responses (52). However, direct evidence linking HMGB1-TLR4 signaling to functional immune activation in BNCT is currently lacking, particularly in clinical settings. Whether HMGB1 interacts with the same receptors or triggers similar immune pathways in BNCT-treated tumors remains unclear and warrants further investigation.

## 4 RT-induced neoantigen presentation

Most current understanding of radiation-induced neoantigen presentation is primarily derived from studies on conventional X-ray RT. Beyond the aforementioned DAMPs, which originate from ICD and are responsible for stimulating Antigen-presenting cell (APC) activation, tumor cell DNA itself acts as a pathogen-associated molecular pattern that is phagocytosed by APCs, particularly DCs (53). This triggers DCs to present tumor associated antigens to CD8^+^ T cells. In the process, IFN-I produced via cGAS-STING signaling promotes DC maturation, thereby enhancing anti-tumor activity (41). RT can also enhance the presentation of neoantigens by CD8^+^ and CD4^+^ T cells, upregulate MHC-II in tumors, and upregulate MHC-I in DCs (54, 55). Apoptosis represents a primary mechanism of radiation-induced cell death (55). RT induces high factor-related apoptosis (Fas) expression in tumor cells, which promotes more effective antitumor responses (56). Apoptotic tumor cells indirectly augment tumor antigens presentation through the release of DAMPs (57). Additionally, factors involved in DNA damage repair processes (such as ATM, RAD51, BRCA1, MUS81, and MRE11) directly or indirectly mediate immune signal transduction (58). However, DNA repair induced by low-LET radiations, such as X-rays and γ-rays, is fast and mainly mediated by the NHEJ pathway, which is a simple repair mechanism. Consequently, their immune-stimulating effects may be weaker than those of high-LET radiations theoretically (59).

In contrast, BNCT, as a high-LET radiation modality, exhibits distinct physical and radiobiological characteristics. However, whether BNCT can induce comparable immunological effects remains largely unclear due to the limited availability of direct experimental evidence. Limited studies have reported that BNCT can promote the release of pro-inflammatory factors and enhance immune cell infiltration, suggesting a potential immunostimulatory role (44, 52); nevertheless, whether these effects are mechanistically analogous to those observed in conventional RT has not yet been established.

Given the immunogenic properties of RT, tumor cell vaccines (TCVs) are considered suitable for combination with RT (22). However, the development of TCVs is hindered by their weak immunogenicity and potential oncogenic risks (45). Although emerging studies have begun to investigate BNCT-based vaccine strategies, current evidence is still limited. Recently, Lv et al. designed a DC-based vaccine in which DCs were educated by tumor cell-derived extracellular vesicles enriched with BNCT-induced DNA fragments, and this vaccine exhibited enhanced anti-tumor immune activation (45). Meanwhile, Yang et al. selected cryopreserved tumor cells as a co-delivery vehicle for tumor antigens and ^10^B in BNCT treatment. The novel boron agent can be internalized by APCs, activating APCs to recognize and present tumor antigens. Their findings suggest that BNCT may contribute to the modulation of the tumor microenvironment toward a more immunologically active state. Additionally, BNCT-induced reactive oxygen species (ROS) may contribute to ferroptosis, thereby promoting tumor cells death (3).

## 5 Immunosuppressive effects and their impact on BNCT efficacy

Although BNCT has been reported to induce immunostimulatory responses, its potential immunosuppressive effects remain poorly characterized. Among these immunosuppressive effects, the recruitment of immunosuppressive cells and the induction of hypoxia are frequently cited in the context of conventional RT. Numerous preclinical studies have demonstrated that interventions targeting these aspects can enhance BNCT efficacy (30, 60). In this section, we will discuss how immunosuppressive cells (MDSCs, Tregs and TAMs) and tumor hypoxia influence BNCT efficacy.

### 5.1 BNCT efficacy can be influenced by MDSCs

MDSCs originate from common myeloid progenitors and act as key suppressors of antitumor immunity (61). They are categorized into three subsets: monocytic MDSCs (M-MDSCs), polymorphonuclear MDSCs (PMN-MDSCs), and early-stage MDSCs (e-MDSCs), the latter is described as progenitors of M-MDSCs and PMN-MDSCs (62). Direct evidence linking MDSCs to BNCT efficacy remains limited. In a clinical study, Chang et al. reported that elevated levels of circulating M-MDSCs prior to BNCT were associated with poorer survival outcomes, suggesting a potential association between MDSCs and treatment response (63). In addition, the same group demonstrated that combining BNCT with a CSF-1 receptor inhibitor significantly prolonged survival in a preclinical model, indicating that targeting myeloid populations may enhance BNCT efficacy (30). However, whether MDSCs play a causal role in BNCT resistance remains to be established.

Most current mechanistic insights into MDSC-mediated immunosuppression are derived from studies on conventional RT. In the context of convention RT, the level of MDSCs is negatively correlated with the efficacy of RT and immunotherapy, as well as patient prognosis (64, 65). RT promotes MDSC recruitment through multiple mechanisms: 1. MDSC-derived mediators (including PGE2, GM-CSF, VEGFA and TGF-β) drive MDSCs recruitment and activation (66). 2. RT directly induces MDSCs accumulation in TME. For instance, tumor-bearing mice subjected to fractionated RT with 2 Gy × 5 exhibited a significant increase in MDSCs after radiation, whereas no such elevation was observed in tumor-free mice, suggesting that RT-induced damage to tumor cells triggers MDSCs recruitment (67). DNA damage activates the cGAS-STING pathway (68). While this pathway is critical for mediating the abscopal effect in anti-tumor immunity (39), it also contributes to immunosuppression, especially via type I IFN (69).In STING knockout mice, tumor radiation resulted in a significant reduction in MDSCs (70). Notably, IFN-β induces tumor cells to express the chemoattractant CCL2, which in turn promotes MDSC accumulation (69). After being recruited into the TME, MDSCs exert potent immunosuppressive functions, mainly through suppressing CD8^+^ T-cell activity (71). Programmed death-ligand 1 (PD-L1) is a key inhibitory immune checkpoint protein that regulates T cell function. Mechanistically, PD-L1 binds to programmed cell death-1 (PD-1) on CD8^+^ T cells, leading to T cell exhaustion and preventing excessive activation (72). MDSCs upregulate PD-L1 expression on their surface to inactivate CD8^+^ T cells and contribute to tumor radioresistance (73). *In vitro*, studies have shown that PD-L1 expression in tumor cells increases significantly 24 hours after radiation. However, abscopal tumor cells do not exhibit such PD-L1 upregulation, indicating that this process may depend on cell-to-cell interactions (74). It has been proposed that CXCL10 is the key regulator of PD-L1 expression in MDSCs (73). Following RT, MDSCs increase CXCL10 transcription, which in turn upregulates PD-L1 expression via the CXCL10/CXCR3 axis (73). MDSCs can also suppress T cell activity by inducing ROS (75). Additionally, arginase-1 activity is also involved in this immunosuppressive process (71). The depletion of L-arginine by MDSCs reduces TCR expression, thereby suppressing T cell activity (76). Other mechanisms of MDSC-mediated immunosuppression include the recruitment of Tregs and the polarization of macrophages from M1 to M2 phenotype (77). However, whether these pathways operate in a similar manner in BNCT remains unclear and requires further investigation.

### 5.2 BNCT efficacy may be influenced by Tregs

To date, there is a lack of direct evidence demonstrating how Tregs influence BNCT efficacy. As key immunosuppressive cells, Tregs suppress anti-tumor immune response within the TME through multiple mechanisms, including secreting immunosuppressive cytokines such as TGF-β and IL-10, as well as depriving IL-2 to inhibit CD4^+^ T-cells activation. Additionally, both TGF-β and IL-10 can enhance the immunosuppressive function of Tregs (78). These findings are largely based on studies in conventional RT.

In the context of RT, Treg responses appear to be dose-dependent. Low-dose radiation (0.075 Gy) mitigates immunosuppression by inhibiting IL-10 production (a cytokine that can activate Tregs), whereas high-dose radiation (2 Gy) stimulates IL-10 secretion (79). However, whether similar dose-dependent effects occur in BNCT remains unknown. Additionally, RT induces TGF-β production in the TME, which promotes Treg recruitment and accumulation, thereby increasing their numbers and suppressive activity (80). Collectively, these effects enhance Treg-mediated immunosuppression and impair tumor treatment efficacy. While targeting Tregs has yielded promising results in optimizing conventional RT efficacy (81). Nevertheless, such promising combinatorial strategies have not yet been reported in BNCT to date. Therefore, the relevance of Treg-mediated immunosuppression in BNCT remains to be clarified.

### 5.3 BNCT efficacy may be influenced by macrophages

Macrophages are generally categorized into two phenotypes: M1 and M2. M1 macrophages exhibit tumor-killing properties, whereas M2 macrophages (referred to as TAM) promote tumor progression (82). More importantly, M2 macrophages secrete chemokines that recruit Tregs and MDSCs into the TME (83, 84). Limited evidence suggests that macrophages may be involved in BNCT-associated immune responses. In a preclinical study, breast cancer-bearing mice were treated with BNCT, and cytokine levels were measured before and after irradiation. Post-irradiation, significant increases in IL-12 and decreases in IL-10 were observed (85). IL-12 is primarily secreted by M1 and exerts a positive role in anti-tumor immune responses, whereas IL-10 is mainly produced by M2 and inhibits such responses (86). This finding suggests that BNCT may also enhance anti-tumor immunity, which is associated with M2-to-M1 macrophage polarization. However, direct evidence confirming this effect is still lacking.

Mechanistic understanding of macrophage polarization in response to radiation is primarily derived from conventional RT, where dose-dependent effects have been reported. A systematic review demonstrated that moderate-dose irradiation (2 Gy × 5) enhances the pro-inflammatory phenotype of M1 macrophages, while high-dose irradiation (>10 Gy) expands the population of M2-like TAMs (87). While the dose-dependent immune polarization pattern observed in conventional RT remains unverified in BNCT, the distinct physics of high-LET radiation suggests a potential divergence. High-LET particles in some contexts are more possible to induce necrotic cell death, which triggers the leakage of intracellular “danger signals” that stimulate innate immune responses (34, 88). It has been hypothesized that BNCT-induced DAMP release may influence macrophage activation; however, whether this leads to macrophage polarization remains unclear and has not been directly demonstrated. Beyond macrophage polarization, macrophage-mediated tumor clearance is further hindered by the CD47-SIRPα immune checkpoint, which transmits the “don’t eat me” signal on tumor cells and severely impairs phagocytosis of residual tumor cells and cancer stem cells (89). Chen et al. engineered a multifunctional nanoliposome delivery system, DOX-CB@lipo-pDNA-iRGD. On one hand, it leverages the nuclear-targeting property of doxorubicin (DOX) to selectively deliver boron-10 into tumor cell nuclei, ensuring sufficient boron accumulation at the nuclear level for effective BNCT. On the other hand, this nanosystem carries a CD47-targeting CRISPR-Cas9 plasmid, which directly knocks down CD47 expression in glioblastoma cells, thereby restoring macrophage-mediated phagocytosis (90). By combining nuclear-targeted BNCT with CD47 gene editing, this integrated strategy markedly enhances anti-tumor efficacy and suppresses tumor recurrence in glioblastoma. *In vivo* data showed that all mice in the control and BNCT monotherapy groups died within 30 days. CD47 knockout alone yielded limited tumor control, with a survival rate below 50% at 66 days. In sharp contrast, the combination group of nuclear-targeted BNCT and CD47 gene editing achieved nearly 100% survival at 66 days (90). Thus, integrating BNCT with CD47 blockade-mediated macrophage activation represents a potential strategy to optimize the therapeutic outcomes of BNCT.

The efficacy of BNCT is directly determined by the T/N and T/B ratio, with higher ratios corresponding to better therapeutic outcomes. Macrophages are proposed as promising carriers for boron drugs due to their inherent properties, including the ability to phagocytose foreign particles, low immunogenicity, biocompatibility, biodegradability, a long half-life, the capacity to cross biological barriers, tropism for hypoxic regions, and tendency to accumulate in the TME (10). However, to date, no animal studies utilizing macrophages as boron drug carriers have been reported.

### 5.4 BNCT efficiency can be influenced by hypoxia

Hypoxia is a common characteristic of the intratumoral microenvironment in most rapidly growing malignant tumors (91). It protects tumor cells from radiation-induced damage, thereby contributing to the development of radioresistance in the context of conventional RT (92). The impact of tumor hypoxia on BNCT efficacy remains insufficiently understood. Limited evidence suggests that hypoxia-related factors may influence BNCT response; however, direct experimental studies are scarce. In a preclinical model, disruption of hypoxia-inducible factor-1α (HIF-1α), was reported to increase expression of the boron transporter SLC7A5 (LAT1), thereby enhancing sensitivity to BNCT, indicating a potential link between hypoxia signaling and BNCT efficacy (60). HIF-1α is a transcription factor crucial for tumor cell survival under hypoxic stress (93). In hypoxic environments, HIF-1α forms a heterodimer with HIF-1β to form the transcription factor HIF1, which binds to hypoxia-responsive elements in target genes to regulate oxygen-dependent gene expression. Through these pathways, HIF-1α upregulates molecules involved in DNA repair (94), increases glucose transporter-1 and induces glycolysis (95).

Most current understanding of hypoxia-induced radioresistance is derived from conventional RT. In the hypoxic environment, radiation fails to induce sufficient ROS (which mediate DNA damage), thus weakening the efficacy of oxygen-dependent RT (96). Notably, hypoxia not only impairs radiation-induced DNA damage but also promotes DNA repair processes. For instance, under hypoxic conditions, sulfhydryl-group-containing substances can reduce damaged DNA, thereby preventing further DNA injury (97). Beyond these physicochemical effects, tumor cells undergo multiple biological adaptations in response to hypoxia, which also enhance radioresistance. A key adaptive change is the upregulation of HIF-1α (93). HIF-1α exerts immunosuppressive responses within the TME through a complex network of mechanisms. First, HIF-1α upregulates vascular endothelial growth factor (VEGF), which not only promotes tumor angiogenesis but also contributes to immunosuppression in the TME (98). VEGF induces immunosuppression by recruiting MDSCs and Tregs, and impairing the antigen presentation capacity of DCs (99). Second, HIF-1α stimulates the secretion of immunosuppressive cytokines such as TGF-β, IL-10, and VEGF, which in turn facilitate the recruitment of immunosuppressive cells and suppress the activity of CTLs, NK cells, and DCs (93). Thus, disrupting HIF-1α expression or alleviating tumor hypoxia may improve RT efficacy. Importantly, HIF-1α can also suppress p53 expression in irradiated tumor cells to reduce apoptosis (100).

In solid tumors, newly formed blood vessels exhibit abnormal structure and function, which induce immunosuppressive effects (101). The impaired perfusion capacity of tumor blood vasculature contributes to the formation of a highly hypoxic TME. Additionally, tumor-associated endothelial cells express reduced levels of ICAM-1 and VCAM-1, which promotes endothelial anergy and impairs the trafficking of immune effector cells into tumors (102). Given the immunosuppressive effects of abnormal vessels, normalizing these aberrant vessels may help restore immune cell function. RT can remodel vascular structure. Emerging vessels are particularly sensitive to RT due to the rapid proliferation rate of their endothelial cells (17). Changes in vascularization following irradiation are dose-dependent (101). Klug et al. demonstrated that neoadjuvant local low-dose gamma irradiation (single dose of 0.5–5 Gy) caused normalization of aberrant vasculature and enabled efficient recruitment of tumor-specific T cells in human pancreatic carcinomas (103). Conversely, a single high dose (>10 Gy) may hinder this normalization process. While high-dose irradiation destroys blood vessels, alters tumor oxygenation levels, and indirectly induces cell death, the resulting vascular damage exacerbates local hypoxia (17), rendering tumor cells more prone to developing radioresistance. However, whether these dose-dependent vascular effects are applicable to BNCT remains unclear.

In contrast to conventional RT, BNCT-induced DNA damage is generally considered to be less dependent on oxygen due to its high-LET characteristics (2). However, this assumption is largely based on radiobiological principles, and its relevance *in vivo* remains to be fully established. Meanwhile, from the perspective of boron delivery BPA may exhibit insufficient accumulation in hypoxic tumor regions, which may impair the efficacy of BNCT against hypoxic tumor cells (60). Thus, BNCT therapeutic efficacy remains to be investigated in hypoxic TME.

It has been proposed that BNCT may differentially affect tumor-associated vasculature due to its tumor-targeting properties; however, direct experimental evidence supporting this hypothesis is currently lacking. Tumor-associated endothelial cells have been reported to express higher levels of LAT1, which serves as a primary transporter for BPA, suggesting that these cells may be more exposed to BNCT-induced damage compared with normal vasculature (104, 105). Nevertheless, whether this differential targeting translates into functional vascular remodeling remains unclear. From a radiobiological perspective, the high-LET particles generated in BNCT produce highly localized energy deposition with a limited path length (5–9 μm), which may confine damage to boron-containing cells. This spatial selectivity raises the possibility that BNCT could influence tumor vasculature without extensively damaging surrounding normal tissues. However, whether BNCT can induce vascular normalization or improve tumor oxygenation has not yet been demonstrated.

## 6 ICI and CAR T-cell therapy: promising combinations with BNCT

The emergence of ICI and CAR T-cell therapy has ushered in a new era of immunotherapy (20, 106). Although combinations of immunotherapy and conventional RT have demonstrated promising clinical outcomes, the potential of integrating these strategies with BNCT remains to be fully explored (107, 108). In this section, we discuss the potential and advantages of combining these modalities with BNCT.

### 6.1 ICIs: a promising partner of BNCT

In conventional RT, the application of ICIs not only enhances the therapeutic efficacy of RT but also helps overcome tumor cell resistance. Among ICIs, anti-PD-1, anti-PD-L1, and anti-CTLA-4 agents are most commonly used in combination with RT. Recently, numerous clinical trials have been investigating RT combined with ICIs for various malignancies (20, 109). Abyaneh et al. demonstrated that combining ICIs with standard treatment modalities represents a promising approach for improving survival and pathological responses in patients with locally advanced esophagogastric adenocarcinoma (110). In another study, Wang et al. evaluated the efficacy of low-dose RT (15 Gy in 5 fractions) combined with a PD-1 inhibitor in a murine small-cell lung cancer model, and this combination significantly induced anti-tumor immune responses and prolonged overall survival in mice (111).

Notably, combinations of BNCT with PD-L1-targeted therapies have been reported (4, 18). In a preclinical study, the first reported combination of BNCT and anti-PD-L1 significantly suppressed tumor growth and enhanced T cell infiltration and activation at tumor sites (112). Fujimoto et al. compared the immune responses and therapeutic efficacy between BNCT alone and BNCT combined with anti-PD-1 (B-NIT). They demonstrated that B-NIT resulted in greater intratumoral CD8^+^ T‐cell infiltration, higher serum HMGB1 levels, and more significant tumor growth inhibition (112). Chiu et al. observed similar outcomes: the combination of anti-PD-L1 and BNCT prolonged tumor growth delay to 6.6 days, compared to 5.4 days with BNCT alone (18). Notably, both studies administered ICIs after BNCT. Additionally, a multifunctional boron agent conjugated with anti-PD-L1 has been reported (4).

Recently, Deng et al. synthesized a ^10^B-containing copolymer (^10^B/siPD-L1) composed of three segments: a cRGD-terminated polyethylene glycol PEG segment (cRGD-PEG), a middle segment with thiol pendant groups, and a ^10^B containing segment with benzoborazole pendant (PBOB). The PBOB segments facilitate the loading of PD-L1 siRNAs. Notably, the cRGD moiety significantly enhances the tumor-targeting ability. The ^10^B/siPD-L1 copolymer rapidly disassembles in response to high levels of glutathione (GSH) and ATP in tumor cells. In contrast, in normal cells with low GSH and ATP levels, ^10^B/siPD-L1 disassembles more slowly and is expelled via exocytosis (4). These properties enable the agent to retain in tumor cells for longer periods while avoiding damage to normal cells. The researchers also demonstrated the immune-activating effects of this novel agent. Interestingly, in mice treated with ^10^B/siPD-L1 followed by neutron irradiation, tumor volume increased slightly in the first 8 days. After day 10, however, tumor volume gradually decreased and remained stable. Compared with BNCT monotherapy, the combination treatment demonstrated improved tumor control (4). These observations suggest a potential therapeutic effect of the combination strategy. Although reports on the synergistic interaction between BNCT and ICI remain limited, considering the critical role of immunosuppressive effects in radioresistance highlights the need to further explore the potential relationship.

### 6.2 CAR T-cell therapy: a promising combination with BNCT

CAR T-cell therapy has emerged as a novel and highly effective treatment modality. Its fundamental principle involves transducing T cells with a CAR to redirect their antigen specificity toward a defined tumor-associated antigen (101). While CAR T-cell therapies have been successfully applied in the management of hematological malignancies, their clinical utility in solid tumors is hindered by several limitations, including impaired tumor homing, an immunosuppressive tumor microenvironment, heterogeneous expression of tumor antigens, limited vascularization, and tumor hypoxia (113).

In the context of conventional RT, several studies have shown that irradiation can modulate the TME and enhance T-cell infiltration through mechanisms such as upregulation of adhesion molecules and induction of pro-inflammatory cytokines (101, 114). These findings provide a rationale for combining RT with CAR T-cell therapy. For instance, intercellular adhesion molecule 1 (ICAM-1) is a cell surface glycoprotein and adhesion receptor primarily known for regulating leukocyte recruitment from circulation to inflammatory sites (115). A previous study demonstrated that ICAM-1 and vascular cell adhesion molecule 1 (VCAM-1) synergistically mediate leukocyte adhesion to endothelial cells in a rat model of colitis (116). Importantly, RT can induce the expression of ICAM-1 and VCAM-1 in lymphatic vessels, facilitating T-cells extravasation and enhancing T-cell infiltration into the TME (117). Hence, this radiation-induced upregulation of adhesion molecules is conducive to CAR T-cell homing to tumors. Additionally, RT-induced upregulation of proinflammatory cytokines remodels the TME from an immunosuppressive “cold” tumor to an immune-inflamed “hot” tumor, which is an environment more favorable for CAR T-cell-mediated tumor killing (109, 114). Furthermore, RT initially eliminates immunosuppressive cells such as MDSCs (30), potentially protecting CAR T cells from early depletion. Low-dose RT (2 Gy) may also normalize tumor vasculature and alleviate hypoxia, thereby mitigating immunosuppressive effects, and normalized vasculature could, in turn, promote CAR T-cell accumulation (113). However, whether these mechanisms are applicable to BNCT remains unclear due to the limited availability of direct evidence.

BNCT differs from conventional RT due to its tumor-targeting capability and high-LET radiation (2). These properties suggest a potential basis for combination with CAR T-cell therapy. In the TME, extracellular matrix (ECM) components and fibroblasts form physical barriers around neoplastic cells, impeding CAR T-cell infiltration (113). The high LET radiation of BNCT may disrupt dense ECM structures, potentially facilitating CAR T-cell penetration through physical barriers. Nevertheless, this characteristic of BNCT remain unclear. Conventional RT may induce lymphopenia, which has been associated with an increased risk of mortality (118). To avoid this potential adverse side effect, CAR T-cell therapy is typically administered after RT. BNCT may reduce off-target damage to surrounding normal tissues due to its selective boron accumulation, although its impact on systemic immune function requires further investigation.

The development of novel boron agents is conducive to the combining with CAR T-cell therapy. Nanotechnology enables boron agents to carry molecularly targeted drugs, such as ICIs, VEGF inhibitors, and HIF-1α inhibitors. These multifunctional boron agents may have potential to enhance tumor targeting, sustain anti-tumor immunity in the TME and alleviate hypoxia. With such promising prospects, an optimal multifunctional boron delivery system may be developed to overcome most obstacles to the combined application of CAR T-cell therapy and BNCT. Crucially, the ‘Trojan horse’ strategy, using CAR T-cells as active vehicles for boron delivery, has been proposed as a potential strategy to address the delivery limitations of traditional boron agents. Previous study has demonstrated that boron-containing nanoparticles can be efficiently internalized by T cells via mechanisms such as electrostatic interactions and clathrin-mediated endocytosis, without significantly impairing their viability or functional properties. Demichelis et al. has demonstrated that tumor-infiltrating lymphocytes can effectively transport boron carbide (B_4_C) nanoparticles into the deep parenchyma of solid tumors, significantly enhancing the T/N boron concentration ratio required for subsequent BNCT (119). Building upon these findings, it is hypothesized that CAR T-cell could serve as potential delivery vehicles, potentially enabling sufficient boron accumulation in poorly vascularized and hypoxic tumor regions that are less accessible to small-molecule agents. However, it should be noted that the tumor infiltration capacity of CAR T-cells in solid tumors remains a significant challenge, and further studies are required to validate their effectiveness as boron carriers *in vivo*. Additionally, it may be possible to engineer boron-containing platforms to co-deliver T cell–recruiting chemokines, thereby enhancing CAR T-cell infiltration and retention within tumors, although this concept remains speculative and warrants experimental investigation. Overall, while the combination of BNCT and CAR T-cell therapy is conceptually attractive, current evidence remains limited. Further studies are needed to determine whether BNCT can effectively modulate the TME to support CAR T-cell function and to evaluate the feasibility and safety of such combinatorial strategies.

## 7 Discussion

**Figure 3:**
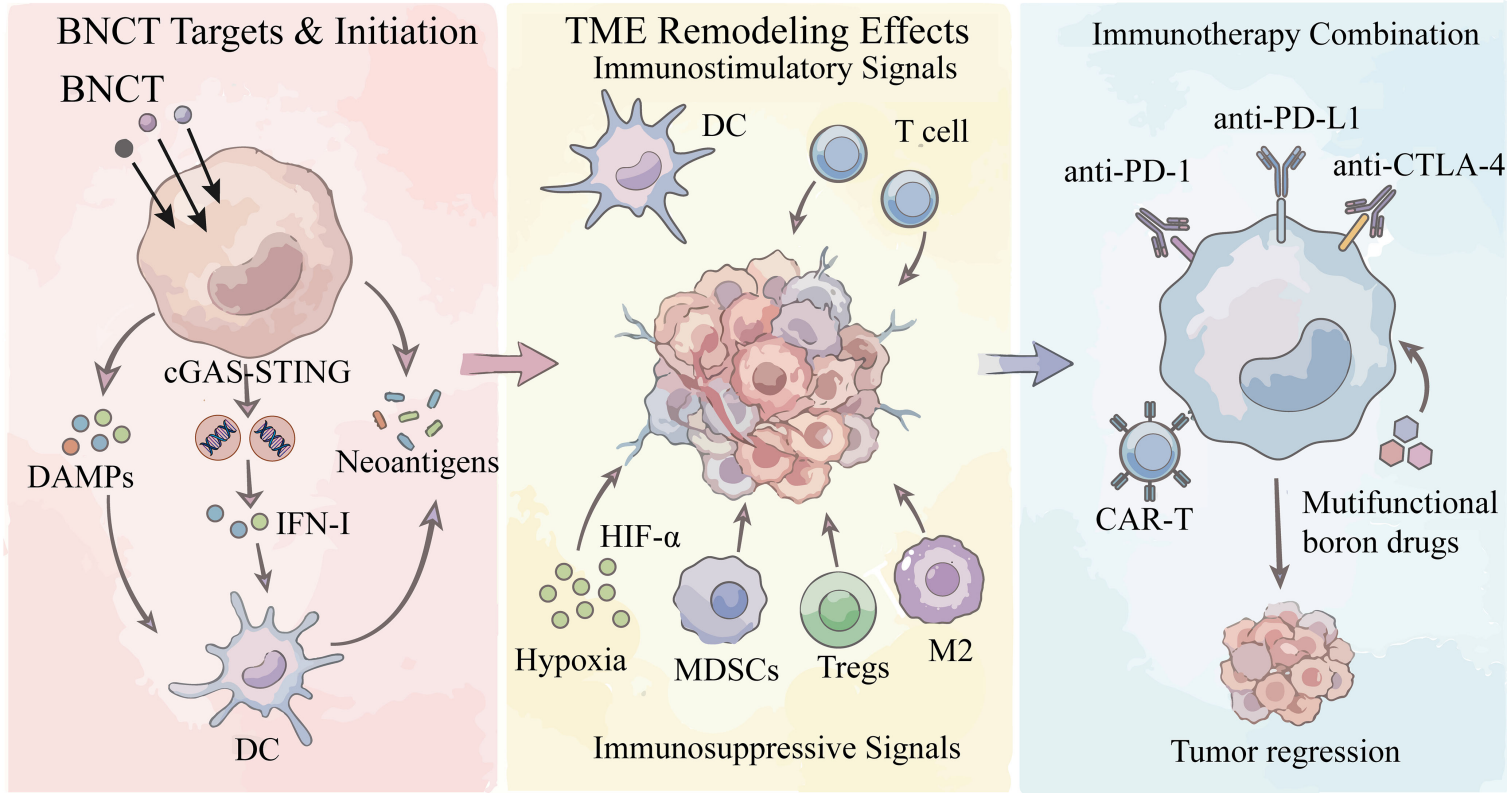
Schematic overview of this article. BNCT selectively targets ^10^B-enriched tumor cells, triggering the release of DAMPs and neoantigens in the TME. Subsequent DNA damage activates the cGAS-STING signaling pathway, driving IFN-I production and DC maturation to initiate anti-tumor immune responses. BNCT remodels the TME, generating both immunostimulatory and immunosuppressive signals. Immunostimulatory signals induces the activation of DCs and T cells. Immunosuppressive signals promote the infiltration of MDSCs, Tregs, M2-type macrophages, and the formation of a hypoxic environment. Combinatorial approaches, including ICIs (anti-PD-1, anti-PD-L1, anti-CTLA-4), CAR-T cell therapy, and multifunctional boron-based drugs, are utilized to counteract TME immunosuppression and synergize with BNCT, thereby achieving robust synergistic tumor regression.

This review summarizes the potential mechanisms of BNCT in remodeling TME and its prospects for combination with immunotherapy. **Figure 3** shows an overview of the key content presented in this article. Available evidence suggests that BNCT-induced ICD may play a crucial role in TME remodeling. While BNCT activates anti-tumor immunity and promotes immune cell infiltration, it also triggers the recruitment of immunosuppressive cells into the TME, contributing to the development of radioresistance. MDSCs, in particular, not only directly suppress anti-tumor immunity by upregulating PD-L1 but also recruit other immunosuppressive cells such as M2-type macrophages and Tregs. Additionally, tumor hypoxia drives radioresistance: the hypoxic TME significantly impairs the therapeutic efficacy of radiation, and hypoxic tumor cells secrete immunosuppressive cytokines to weaken T cell function. Targeting these immunosuppressive components thus holds promise for optimizing BNCT outcomes. Furthermore, we highlight that ICIs and CAR T-cell therapy are promising candidates for combination with BNCT. Overall, this is the first review to systematically discuss the immunomodulatory effects of BNCT, with the aim of providing future directions for BNCT development.

Recently, single-cell RNA sequencing analyses provide compelling evidence that BNCT remodels the TME at a cellular level (44). Specifically, BNCT enhances DC antigen presentation, promotes CD8^+^ T-cell activation, and increases NK-cell cytotoxicity. These observations are consistent with BNCT-induced ICD and support the activation of innate immune sensing pathways. Bulk transcriptomic studies further confirm that therapeutic-dose BNCT triggers early NF-κB dependent inflammatory responses in tumor cells, leading to significant secretion of GM-CSF, a key cytokine that recruits and activates immune cells in the TME (120). Notably, BNCT appears to preserve immune cell viability while selectively eliminating tumor cells, in contrast to conventional photon-based RT. This immune-preserving property may shift the balance toward sustained anti-tumor responses and partly explain the observed abscopal effects and immune memory (44). However, current evidence is largely derived from preclinical models, and further validation in clinical settings is required.

Despite these promising preclinical findings, the combination of BNCT with immunotherapy requires further refinement. The exploration of BNCT’s immunomodulatory mechanisms and synergistic potential with immunotherapy in this review is largely built on extrapolations from well-characterized conventional RT principles, a necessity stemming from the relative paucity of direct evidence specific to BNCT. Although BNCT shares the same fundamental biological effects with conventional RT, including DNA damage induction and ICD initiation, as a novel RT modality, its high-LET radiation and tumor-targeted cytotoxicity may present potential divergences in immune regulation. Notably, there is evidence that carbon ion irradiation (CIR) can induce a stronger anti-tumor immune response and greater activation of the cGAS-STING pathway compared to photon irradiation, which is largely attributed to differences in linear energy transfer (121). While the cGAS-STING pathway is widely implicated in mediating BNCT’s immunostimulatory effects, whether BNCT exhibits distinct characteristics relative to other high-LET radiotherapies remains unclear. Clinically, in malignant melanoma, BNCT has been associated with prolonged survival at lower radiation doses relative to CIR (122, 123), supporting potential differences in therapeutic responses between BNCT and CIR in certain tumor types. We hypothesize that these differences in anti-tumor responses may be attributed to the specific properties of boron-containing drugs or BNCT’s highly tumor-targeted cytotoxicity. Therefore, clarifying the mechanistic differences between BNCT and other high-LET radiotherapies represents a critical priority for advancing BNCT-based combination strategies.

The balance between BNCT-induced immunostimulatory and immunosuppressive effects within the tumor microenvironment is complex. While the application of PD-1/PD-L1 inhibitors or CSF-1 receptor antagonists may optimize BNCT outcomes, the efficacy of such combination therapies remains clinically uncertain. A major hurdle is the absence of standardized protocols for BNCT irradiation dosing and fractionation strategies. Additionally, the optimal timing of immunotherapy administration (whether sequential or concurrent) to maximize the synergistic effects with BNCT remains undefined. Notably, the optimal immunotherapy schedule appears to vary with the type of boron agent used in BNCT-based combination strategies. Specifically, Fujimoto et al. adopted a sequential strategy of anti-PD-1 antibody administration prior to BNCT (112), whereas Chiu et al. employed the reverse strategy, delivering anti-PD-1 therapy subsequent to BNCT (18). Notably, Karapetyan et al. previously emphasized that treatment scheduling is a critical determinant of the efficacy of radioimmunotherapy combinations (109). Radiation dose represents an additional critical challenge: different biological effects are observed with low-dose versus high-dose radiation. Jarosz-Biej et al. demonstrated that high-dose radiation (>10 Gy) can induce hypoxia and promote immunosuppressive effects, whereas intermediate-dose radiation (4–10 Gy) triggers cancer cell death without eliciting immunosuppression (17). Notably, radiation’s effects on immunosuppressive cell abundance and ICD induction also appear to be dose-dependent (21, 124).

The translation of dose-dependent immune and vascular responses from conventional RT to BNCT is complicated by the unique dosimetry of neutron capture reactions. Unlike the relatively uniform dose deposition of photon beams, BNCT dosimetry relies on the compound biological effectiveness (CBE) factor, which integrates the heterogeneous LET and relative biological effectiveness (RBE) of the emitted high-LET particles (125). The CBE factor is inherently tissue and agent-specific, reflecting the microscopic distribution of boron delivery agents (e.g., BPA vs. BSH) within distinct cellular and tissue compartments (126). This spatial heterogeneity suggests that a single physical dose in BNCT can lead to divergent biological effects depending on local boron concentration. For instance, whereas conventional RT relies on a defined moderate-dose (2 Gy × 5) to optimize M1 macrophage polarization (67), the high-LET particles generated in BNCT may elicit robust pro-inflammatory signals via clustered DNA damage, even at low total absorbed doses, provided that the T/N boron concentration ratio is sufficiently optimized. Therefore, the threshold for immune-stimulating or suppressive effects in BNCT is likely defined by boron micro-distribution rather than gross physical dose, highlighting the need for more sophisticated radiobiological models to predict therapeutic outcomes. Another significant translational barrier in BNCT is the heterogeneous distribution of boron agents within the TME. Current clinically utilized boron drugs, such as L-BPA, rely on LAT1 for cellular uptake (19). However, the expression of LAT1 is highly variable among tumor cells. As a result, tumor cells with subtherapeutic boron concentrations fail to receive an effective radiation dose and thus evade elimination. This provides a potential rationale for combining BNCT with immunotherapy: although BNCT potently eradicates boron-rich tumor cells and induces localized ICD, it leaves behind residual tumor cells with insufficient boron accumulation. Combining BNCT with ICIs or CAR-T cell therapy can amplify the systemic anti-tumor immune response initiated by BNCT-induced local ICD, potentially enabling the immune system to recognize and eliminate these boron-deficient residual tumor cells. In this context, immunotherapy serves as a crucial systemic strategy, compensating for the intrinsic spatial limitations of boron drug delivery and preventing local recurrence.

Nevertheless, while systemic immunotherapy offers a powerful solution to boron heterogeneity, the potential overlapping toxicities between BNCT and immunotherapy must be considered. The released ICD and pro-inflammatory cytokines may induce severe acute local inflammatory responses. Furthermore, the administration of ICIs can lead to excessive T−cell hyperactivation, thereby increasing the risk of immune−related adverse events. Theoretically, combining BNCT and immunotherapy could trigger these autoimmune cascades. To mitigate these risks, exploring optimal treatment sequencing and developing TME-responsed multifunctional boron drugs are crucial.

## 8 Conclusion

BNCT is an interdisciplinary treatment modality integrating nuclear physics, chemistry, biology, and medicine, among other fields (127). While preclinical studies have demonstrated encouraging synergistic effects between BNCT and immunotherapy, their clinical translation is still hindered by several fundamental and interrelated challenges.

Firstly, the therapeutic efficacy of BNCT is intrinsically determined by the distribution of boron at the cellular and subcellular levels, making intratumoral heterogeneity a primary bottleneck (128). Although criteria for ideal boron delivery agents have been proposed (19), clinically available compounds such as BPA and BSH fail to simultaneously satisfy the requirements of tumor selectivity, retention, and uniform distribution. This limitation directly translates into dosimetric uncertainty and heterogeneous biological responses. Therefore, we propose that the development of next-generation boron carriers—particularly those with tumor-targeting and TME-responsive properties—represents a prerequisite for advancing BNCT-based combination therapies.

Secondly, the integration of BNCT with immunotherapy necessitates a truly personalized treatment paradigm. On one hand, the immunological consequences of BNCT, including ICD, are dynamic, and the therapeutic benefit of immunotherapy may depend on the timing of administration relative to these immune events. However, robust biomarkers capable of quantifying ICD and guiding optimal treatment scheduling remain lacking. On the other hand, interpatient variability in boron accumulation further implies that each patient may possess a distinct “biological window” for maximal therapeutic benefit (129). In this context, advanced imaging modalities such as PET-based boron tracing offer a promising strategy to quantitatively assess boron distribution and guide patient-specific treatment planning. We argue that the convergence of functional imaging and immune monitoring will be essential for defining this individualized therapeutic window.

Finally, the widespread adoption of BNCT has historically been constrained by the substantial capital investment required for neutron source infrastructure. In line with the prevailing consensus in the BNCT community, the development of accelerator-based neutron sources has been a critical driver for improving the feasibility, safety, and accessibility of BNCT, thereby facilitating its transition from reactor-based systems to hospital-based clinical applications (130, 131). From an economic perspective, direct comparisons between accelerator-based BNCT and other particle therapies, such as proton or carbon-ion RT, remain limited. Importantly, such comparisons should consider not only infrastructure costs but also differences in treatment paradigms, as BNCT is often delivered in a single or limited number of fractions, whereas particle therapies typically involve multiple fractions (132, 133). Therefore, definitive conclusions regarding comparative cost-effectiveness remain premature and will require modality-specific analyses incorporating real-world utilization, clinical outcomes, and long-term healthcare resource consumption.

In summary, the future success of BNCT-immunotherapy combinations will depend on bridging the gap between localized physical dose deposition and systemic immune activation through innovations in drug delivery, biomarker development, and treatment personalization. Only by addressing these interconnected challenges can BNCT evolve from a niche modality into a cornerstone of precision oncology.
